# Knowledge and Practice of and Attitude Toward Epidural Analgesia Among Pregnant Women in Jazan Region of Saudi Arabia

**DOI:** 10.7759/cureus.25828

**Published:** 2022-06-10

**Authors:** Mohammed Ageel, Abdullah Shbeer, Abdullah Dahdoh, Almoayad Makrami, Khalid Alhazmi, Dhaifallah Zaeri, Hatim Mutanbak, Alwaleed Alhazmi

**Affiliations:** 1 Surgery, Jazan University, Jazan, SAU; 2 Medicine, Jazan University, Jazan, SAU

**Keywords:** awareness, knowledge, pregnancy, women, epidural analgesia

## Abstract

The aim of this study was to assess the knowledge, sources of knowledge, attitudes (fears and misconceptions) toward epidural analgesia (EA), and practices of parturient delivery in the Jazan region of Saudi Arabia. A cross-sectional study was conducted using a self-administered survey questionnaire distributed in the antenatal care clinics of the obstetric departments of major hospitals. A total of 454 women participated in this study. Of the participants, 219 (48%) belonged to the 31 to 40-year age group and 134 (30%) to the 21 to 30-year age group. Most participants (344, 76%) had a bachelor's degree. The prevalence of epidural catheter use was 23.6% among pregnant women. Statistically significant differences in educational level and residence were found between the women (*p* < 0.001). The two most common sources of information cited by the pregnant women were healthcare staff and family and friends. The most common motive reported by women was to relieve labor pain effectively, and the most frequently cited barriers preventing women from receiving EA were the possibility of injury to important organs and the inability to walk after EA. The present study demonstrates a low level of knowledge about EA among pregnant women in the region. More awareness and guidance about EA are warranted.

## Introduction

Women experience severe pain during labor. The contractions of the uterine muscles and the pressure on the cervix create pain during labor. Strong cramping, as well as an achy feeling, can be felt in the abdomen, groin, and back. Some women may get pain in their sides and thighs. Other causes of pain during labor include the baby's head pressing on the bladder and bowels, as well as the delivery canal and vaginal stretching [1-2]. In most cases, women dread this pain but choose to endure it altogether. Some women take pride in enduring labor pain despite the intensity to show strength. On the contrary, other women cannot withstand this pain but have to weather it because of the culture in which they live [3-4].

Several analgesic techniques are used to attain pain-free labor, including epidural analgesia (EA). EA is performed by inserting an indwelling catheter into the epidural space, after which the patient receives continuous or multiple infusions of local anesthetics and opioids [5]. The need for EA is significant to avoid other adverse outcomes of labor pain when giving birth such as hypertension, longer labor time, and postpartum depression [6-7]. The immediate result of EA is pain relief during labor and delivery [8]. In developed countries, more women appreciate the value of EA and ask for it upfront [9-10]. This is not the case for women in the Jazan region of Saudi Arabia.

Women in the Jazan region of Saudi Arabia have little knowledge about EA because of cultural practices that encourage them to endure labor pain. Women in this region do not understand the beneficial outcomes of using EA during labor pain. Gari et al. illustrated that women will shy away from EA in most cases because they do not understand what it entails [5]. This shows the need to sensitize both men and women in the Jazan region about the benefits of EA. This study is critical in helping understand the level of knowledge available in the population and in guiding practitioners as they disseminate more information to women.

Remarkably, the existing attitude toward labor pain in the Jazan region of Saudi Arabia is that it is a vital part of womanhood. These women's pride in enduring labor pains is the main reason for their negative attitudes toward EA. They primarily lack sufficient knowledge to make informed decisions [11]. Nonetheless, those who know about EA choose not to use it because of preconceived notions about pain relief during labor pains. Women in the region do not regard pain relief during labor as a sign of being a strong or even good woman. Overall, this skepticism is a result of the fear of societal judgment [5]. This is particularly true for those with lower literacy levels.

This paper presents statistics about the knowledge, attitudes, and practices concerning EA during labor pain. The research outcomes will be helpful for future studies and professional use by medics as they sensitize populations about the benefits of EA. The primary aim of this study was to establish best practices for medical practitioners regarding women in labor and how to address their fears of pain relief. The overarching fear of adverse outcomes resulting from the injection of EA during labor pain must be addressed [11]. Doing so will help rejuvenate the enthusiasm of women for pain relief during labor, enabling them to enjoy the benefits of reducing blood pressure and oxygen utilization.

Studies on women's and health-care providers' knowledge and misconceptions and comparing their level of awareness with multiple factors are important. These will help in better understanding the issue and educate health educators on how to handle pregnant women, address their fears, provide knowledge, and correct common misconceptions. Providing education about EA and the options and availability of effective pain relief for labor to expectant mothers is vital. However, little information is known, especially in the Jazan region, about the knowledge of women and healthcare providers about EA and the factors determining acceptance rates, which vary depending on various factors, including geography, education, and finance [5].

The aim of this study was to assess the knowledge, sources of knowledge, attitude toward EA, and practices of parturient delivery in the Jazan region of Saudi Arabia. Several comparable studies have been conducted worldwide [12-14]. Three similar studies were conducted in Saudi Arabia, in Riyadh [10], Jeddah [5], and Khamis Mushait [8]. Therefore, this study was conducted in Jazan to fill the knowledge gap.

## Materials and methods

Study area and population

The study was conducted in the antenatal care clinics of the obstetric departments of five major hospitals in the Jazan region of Saudi Arabia. Jazan region is located in the southwest corner of Saudi Arabia and directly north of the border with Yemen. The target population for this study is pregnant women from the Jazan region. This study was approved by the ethical committee at Jazan University.

Study design, sampling method, and sample size

The study used a cross-sectional design. Data were collected using a self-administered questionnaire prepared in a previous study regarding awareness, attitude, and desire for labor analgesia. A convenience sampling design was used to recruit participants. The sample size of the study was 440 pregnant women, which was estimated using the sample size formula for cross-sectional studies based on a p-value of 50%, a 95% confidence interval, and an error rate of less than 5%.

Inclusion criteria

This study included all pregnant women who visited antenatal care clinics during the data collection period (February 2021 to June 2021). Females who were not pregnant and those who refused to participate were excluded.

The questionnaire

An organized, self-administered questionnaire was used to collect the data, which consisted of four parts. The first part contains the demographic data, including age, education, employment status, monthly income, location of residence, number of pregnancies, history of normal vaginal delivery (NVD), history of cesarean section (CS), and the type of hospital visited for the antenatal care. The second part assesses the knowledge, in which women were asked to respond with “true,” “false,” or “do not know” to the four questions that tapped their opinion/knowledge on EA: whether contractions become weaker with EA, whether the epidural needle insertion is painful, whether EA is convenient and will ease delivery by allowing women to push when needed, and whether EA leads to complications. In addition, the women were also asked about their sources of knowledge about EA. The third part assesses attitude assessment using six items: "have you used epidural anesthesia in labor?", "do you think using epidural anesthesia helps relieve childbirth pain?", "do you think epidural analgesia should be made available for all women in labor if they desire its use?", "have you received adequate information about pain relief in labor including epidural anesthesia?", "if you are seriously considering epidural anesthesia, would you like to know more about it?", "will you order epidural anesthesia during this labor?". The fourth part includes a list of the motives behind using epidural anesthesia and barriers against using epidural anesthesia items, which the pregnant women can select one or more of the items in each list. The motives list includes "to effectively relieve childbirth pain", "to ease the effort during labor", "encouragement from friends or family", "encouragement from healthcare staff", and "previous experience with EA". The barriers list includes "fear of needles (syringes)", "The needle may injure an important organ in the back", "Its use may cause harm to the fetus", "I may not be able to move for more than 1 day after birth", "No motivation from friends or family" and "No motivation from healthcare staff".

A pilot study was conducted to test the feasibility and applicability of the questionnaire. Twenty pregnant women were surveyed and took 10-15 min to complete the questionnaire, their responses were excluded from the analysis of the main data. The questionnaire was a web-based survey using an electronic Google Form. The questionnaire link was distributed to the pregnant women who agreed to participate in this study during their visit to antenatal care clinics via an online social platform (WhatsApp). At the beginning of the questionnaire, consent was required to continue. The data were only available to the research team.

Data analysis

The collected data were analyzed using SPSS version 23 (IBM Corp., Armonk, NY) to obtain descriptive statistics, including frequency, mean, and percentage. Furthermore, the chi-square test was used to compare categorical variables. Also, the relationship between factors of motives behind using EA and barriers against using EA items, and the willingness to order EA during labor (Will you order epidural anesthesia during this labor?) were analyzed by performing multinomial logistic regressions, using the R function “multinom” [15]. We performed this analysis to test whether the above-mentioned factors could be good predictors of the willingness to order EA during labor. The categorical nature of our variables made them suitable for this type of analysis. A p-value of < 0.05 was used to indicate statistical significance.

## Results

A total of 454 pregnant women participated in the study. As shown in Table 1, almost half (219, 48%) of the participants belonged to the 31 to 40-year age group, and 134 (30%) belonged to the 21 to 30-year age group. Most participants (344, 76%) had a bachelor's degree, and 212 (47%) had an intermediate financial status (6,500-13,000 SAR). The pregnant women in this study who lived in an urban setting represented 54% (245 women) of the study population. The prevalence of epidural catheter use was 23.6% among pregnant women. Statistically significant differences in educational level and residence were found between the women (p < 0.001).

**Table 1:** Summary of the demographic characteristics of the participants (N = 454)

Demographic characteristic		Count n (%)	Past labor with EA	p-Value
Age group	<20 years	20 (4.4)	3	0.70
	21–30 years	134 (30)	32	
	31–40 years	219 (48)	55	
	≥41 years	81 (18)	17	
Employment status	Employed	224 (49)	56	0.48
	Unemployed	230 (51)	51	
Educational level	Secondary school or less	83 (18)	12	<0.01
	Bachelor's degree	344 (76)	79	
	Postgraduate degree	27 (5.9)	16	
Monthly income (SAR)	<6,500	130 (29)	26	0.51
	6,500–13,000	212 (47)	52	
	>13,000	112 (25)	29	
Residence	Rural	209 (46)	36	<0.01
	Urban	245 (54)	71	
Number of pregnancies	1 or 2	168 (37)	49	0.12
	3 or 4	169 (37)	34	
	≥5	117 (26)	24	
History of NVD	At least one	376 (83)	72	0.27
	Never	78 (17)	35	
History of CS	At least one	154 (34)	67	<0.01
	Never	300 (66)	40	
Type of Hospital	Public	327 (72)	70	0.02
	Private	127 (28)	37	

As shown in Table 1, among the participants in this study, 78 pregnant women had never had an NVD, whereas 300 pregnant women had never had a history of CS. Furthermore, 77 pregnant women had histories of both NVD and CS. Most pregnant women visited the antenatal care clinics of public hospitals (327, 72%).

**Figure 1:**
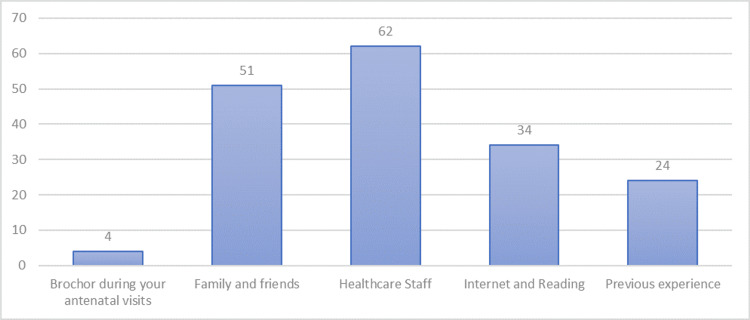
Source of information about EA EA: epidural analgesia

The women's knowledge about EA was examined through the four questions presented in Table 2. Most women responded “Don’t know” to all the questions, which denotes that lack of knowledge was predominant. A higher percentage of pregnant women answered “true” regarding all four statements presented in Table 2. The chi-square test revealed statistically significant differences between responses (p < .001). As shown in Figure 1, the two most common sources of information cited by the pregnant women were healthcare staff, followed by family and friends.

**Table 2:** Knowledge items about EA EA: epidural analgesia

Questionnaire item	TRUE	FALSE	Don’t Know	p-Value
	n (%)	n (%)	n (%)	
Contractions become weak or stop completely after administration of epidural anesthesia	116 (26)	48 (11)	290 (64)	0.001
Epidural insertion is more painful than the labor pain itself	59 (13)	49 (11)	346 (76)	0.001
Epidural anesthesia reduces labor pain and allows the mother to push when needed	137 (30)	31 (7)	286 (63)	0.001
Epidural anesthesia causes serious complications	189 (42)	24 (5)	241 (53)	0.001

Table 3 presents the women's attitudes toward and practice of EA, which indicates that 107 (23.6 %) of the respondents had used EA in labor. However, 64% of the participants believed that EA helps in labor pain relief. Most participants (55%) had not received adequate information on the use and benefits of EA in labor, and 70% would like to know more about it. Only 36% of the participants expressed an intention to use EA during their labor.

**Table 3:** Attitudes and practice toward EA EA: epidural analgesia

Variable	n (%)
Have you used epidural anesthesia in labor?	
Yes	107 (23.6 )
No	347 (76.4 )
Do you think using epidural anesthesia helps relieve childbirth pain?	
Yes	290 (64)
No	54 (12)
Not Sure	110 (24)
Do you think epidural analgesia should be made available for all women in labor if they desire its use?	
Yes	346 (76)
No	49 (11)
Not sure	59 (13)
Have you received adequate information about pain relief in labor, including epidural anesthesia?	
Yes	175 (39
No	250 (55)
Not sure	29 (6.4)
If you are seriously considering epidural anesthesia, would you like to know more about it?	
Yes	318 (70)
No	76 (17)
Not sure	60 (13)
Will you order epidural anesthesia during this labor?	
Yes	162 (36)
No	183 (40)
Not sure	109 (24)

The participants who showed an intention to use EA in labor (162, 36%) were asked about their motivation. As shown in Table 4, the participants' most common motives to order EA were its effectiveness in relieving childbirth pain (92%) and easing their effort during labor (90%). The least common motivation was encouragement from friends or family (70%). On the other hand, participants who were not sure or were unwilling to order EA during labor (292, 64%) were asked about the barriers that prevented them from using EA (Table 5). The most cited barriers were the risk of injury to important organs in the back (81%) and lack of movement for more than one day after birth (74%), whereas the least frequently cited barrier was the risk of harm to the fetus (35%).

**Table 4:** Motives behind using EA EA: epidural analgesia

Motive	n (%)
To effectively relieve childbirth pain	149 (92)
To ease the effort during labor	145 (90)
Encouragement from friends or family	114 (70)
Encouragement from healthcare staff	135 (83)
Previous experience	138 (85)

**Table 5:** Barriers to using EA EA: epidural analgesia

Barrier	n (%)
Is the fear of needles (syringes) a reason that prevents you?	136 (47)
The needle may injure an important organ in the back	237 (81)
Its use may cause harm to the fetus	102 (35)
I may not be able to move for more than one day after birth	216 (74)
No motivation from friends or family	171 (59)
No motivation from healthcare staff	135 (46)

The result of the multinomial logistic regression analysis found that several items of motives and barriers were found to be good predictors of willingness to order EA for the current labor (Table 6). For motives, effectiveness in relieving childbirth pain (0.001), easing the effort during labor (0.043), and encouragement from friends or family (0.001) were significant predictors of willingness to order EA for the current labor. On the other hand, significant predictors that decrease the willingness of pregnant women to order EA were the fear of needles (0.008) and the lack of movement for more than one day after birth (0.014).

**Table 6:** Multinomial logistic regressions omnibus likelihood ratio tests for willingness to order EA for the current labor and motive and barrier items EA: epidural analgesia

Predictor	χ²	df	p
Motives			
To effectively relieve childbirth pain	15.86	2	0.001
To ease the effort during labor	6.29	2	0.043
Encouragement from friends or family	13.12	2	0.001
Encouragement from healthcare staff	5.33	2	0.069
Previous experience	0.52	2	0.768
To effectively relieve childbirth pain	15.86	2	0.001
Barriers			
Is the fear of needles (syringes) a reason that prevents you?	9.77	2	0.008
The needle may injure an important organ in the back	2.40	2	0.301
Its use may cause harm to the fetus	2.06	2	0.357
I may not be able to move for more than 1 day after birth	8.60	2	0.014
No motivation from friends or family	0.03	2	0.985
No motivation from healthcare staff	3.49	2	0.175

## Discussion

This study was conducted in the Jazan region of Saudi Arabia to evaluate the prevalence and knowledge of and the attitude toward EA. Labor is a painful experience, and women have the right to make an enlightened decision regarding EA use [5]. The study sample had the highest number of responses from pregnant women or women who had experienced labor compared with previous studies in Saudi Arabia and other countries that had similar objectives. It involved women of various age groups, types of residence, economic status, work status, and educational levels. The study uncovered that only 23.6% of women had undergone EA, which is lower than that reported in previous studies conducted in Saudi Arabia, such as 32.3% in Khamis Mushait and 32% in Jeddah [5,8]. It also established that the higher the educational level, the higher the frequency of EA use, which contradicts the findings of other studies [16]. Moreover, women in urban cities had a higher prevalence of EA use than those in rural areas [17]. The 31 to 40-year age group had a higher prevalence of EA use than the other age groups, which is consistent with the results reported in the literature [1,18-19]. In Nigeria, research showed that 72% of women would accept EA and 74% would recommend it to their friends and relatives [20]. In Vietnam, women are more skeptical of EA because of misinformation about the procedure [21].

 Approximately 26% of women think that uterine contraction is weakened by EA. This figure is considered high compared with 19.8% in Jeddah and 25.3% in Khamis Mushait and lower compared with 36.5% in Riyadh. Only 30% of the women believed that EA reduces labor pain and allows women to push when needed, compared with 59.8% in Riyadh and 40.6% in Jeddah. When asked about the presence of complications, 42% of the women thought that it can cause serious complications, compared with 38.7% in Khamis Mushait [5,8,22]. These findings are supported by studies from India [14], Nigeria [19,23], Ethiopia [21], and Vietnam [18], where a considerable number of women have myths regarding the effect of EA on uterine contractions.

Approximately 64% of the women in this study had a positive attitude toward EA as a method of pain relief, 24% had an uncertain attitude, and only 12% had a negative attitude. The findings of this study suggest that a lack of sufficient knowledge to make informed decisions is the main reason for the negative attitude. Studies have concluded that the lack of knowledge and women's pride in enduring labor pains are the main reasons for their negative attitudes toward EA [11]. Nevertheless, only 36% of women considered ordering EA in their next delivery, whereas the rest were either uncertain or do not consider it, which is almost the same attitude as that exhibited by women in the UAE. It is a crucial finding which can be used to tailor the approach to such patients and alleviate their concerns. Previous bad experience of pain during normal vaginal delivery significantly reduces the desire to experience NVD without EA [16]. This percentage was higher than that among women in Riyadh (32%) [10,24]. In Nigeria, more attitudes are shifting owing to the availability of positive information, especially among women with higher educational levels [25].

Among women, 76% thought that the availability of EA is necessary; this percentage is lower than that in Khamis Mushait (90.2%) and higher than that in Jeddah (64.3%) [5,8]. In Ludhiana, India, a study found that approximately 96.2% of women had a positive attitude toward EA [26].

The most common motive for having epidural catheters among pregnant women was its effectiveness in relieving childbirth pain (92%), whereas the least common cause was encouragement from friends or family. These findings are contrary to those of a previous study in Riyadh, which attributed the uncertain attitude to the source of motives, mainly friends and relatives [10]. The result of the multinomial logistic regression analysis identified effectiveness in relieving childbirth pain, easing the effort during labor, and encouragement from friends or family as possible predictive motivational factors for willingness to order EA for the current labor.

When women were asked about the barriers against using EA, their biggest fear was the possibility of injury to important organs during catheter insertion (81%). When asked about concerns that the baby will experience side effects due to the procedure, 35% were concerned, which is high compared with 6% in Riyadh. Of the women in this study, 74% were worried that they might lose the ability to walk for a day, which is a relatively high percentage compared with 15.9% in Jeddah, 21.6% in Riyadh, and 34.5% in Khamis Mushait [5,10,12]. Other studies have shown that women are afraid of using EA because of the judgment of society [20,27]. The result of the multinomial logistic regression analysis showed that the fear of needles and lack of movement for more than one day after birth were significant predictors that prevent pregnant women from ordering EA for their current labor.

Some limitations of this study need to be acknowledged. Sociocultural factors are important deterrents, which should be taken into consideration for future study. Many people believe that women are supposed to bear the pain as per their culture. Using EA or alleviating pain might be against the ritual/religious practice [4]. The influence of sociocultural factors may play a key role in the level of knowledge and attitude toward EA and, therefore, should be addressed in such studies.

## Conclusions

The present study demonstrates a low level of knowledge and awareness about EA among pregnant women. More awareness and guidance about EA should be provided to pregnant women in the Jazan region. For this purpose, evidence-based information on EA should be provided during the antenatal period to improve knowledge and attitude. A health education program should be established to provide information about EA to all pregnant women who want to find out more about EA. The educational program should include observation and communication, and a simple diagram of the EA procedure to ease understanding of the coordination between obstetricians and anesthesiologists. Also, efforts should be made to dispel misconceptions and fears about EA.
